# A comparative therapeutic management of anoestrus in buffaloes using insulin and GnRH

**DOI:** 10.14202/vetworld.2015.804-807

**Published:** 2015-06-30

**Authors:** R. D. Purkayastha, S. N. Shukla, O. P. Shrivastava, P. R. Kumar

**Affiliations:** 1 Department of Veterinary Gynaecology & Obstetrics, College of Veterinary Science & Animal Husbandry, Tripura, India; 2 Department of Veterinary Gynaecology and Obstetrics, College of Veterinary Science & Animal Husbandry, Nanaji Deshmukh Veterinary Science University, Jabalpur, Madhya Pradesh, India; 3 Department of Veterinary Gynaecology & Obstetrics, Faculty of Veterinary & Animal Sciences, Banaras Hindu University, Varanasi, Uttar Pradesh, India

**Keywords:** anoestrus, buffalo, buserelin, gonadotropic releasing hormone, insulin

## Abstract

**Aim:**

Anoestrus is one of the most common functional disorders of the reproductive cycle in buffaloes. In spite of technical advancement, there is no single cure for the management of anoestrus. Therefore, the aim of this study was to find out the efficacy of gonadotropic releasing hormone (GnRH) and metabolic hormone for the management of true anoestrus in buffaloes.

**Materials and Methods:**

The experimental animals were selected on the basis of history, gyneco-clinical examinations and progesterone estimation. Deworming was done with Fenbendazole and thereafter mineral mixture was given @ 50 g per animal per day for 10 days in all the selected buffaloes before the start of treatment. The selected buffaloes were randomly divided into four groups (n=25). In Group I, buffaloes were administered 20 µg of buserelin intramuscularly. Buffaloes of Group II were administered long-acting insulin @ 0.25 IU/Kg body weight subcutaneously for 5 consecutive days. In Group III, buffaloes were treated with a combination of insulin and buserelin in the above-mentioned doses whereas buffaloes of Group IV were kept as untreated control.

**Results:**

The higher oestrus induction (64% vs. 28%) was found in Group III and differed significantly (p<0.05) as compared to control group. The conception rate (69.23% vs. 66.66%) was also found higher in Group III but did not differ significantly among the treated groups. The mean time taken for the onset of oestrus was recorded significantly shorter in insulin (8.80±0.69) and GnRH (7.60±0.92 days) alone and as compared to other (Group III, 14.43±0.83 and Group IV, 20.57±1.69 days) groups.

**Conclusion:**

The results of this study indicated better fertility response using Insulin plus Buserelin in true anoestrus buffaloes under field conditions.

## Introduction

Buffalo is considered as black diamond due to its eminent position among the milk producing animals, but their poor reproductive efficiency, mainly because of anoestrus/sub-oestrus, affect economy of the farmers in terms of milk yield, net calf crop and additional cost of rearing. In India, the incidence of anoestrus in buffaloes has been reported from 9% to 85.5% [1-4] and economical losses approximately Rs. 372.90 per day per animal [5].

In contrast to earlier literatures, recent reports indicated that normal follicular recruitment and emergence of follicular waves occurs in true anoestrus buffaloes, but dominant follicle fails to ovulate and ultimately regress probably due to derangement during the critical phase of selection, deviation and dominance [6-8]. The growth and maturation of follicle are under the control of hormones (gonadotropic releasing hormone [GnRH], follicle stimulating hormone [FSH] and luteinizing hormone [LH]) as well as intra-ovarian growth factors.

GnRH causes synthesis and release of FSH and LH which in turn control the gamatogenesis and steroidogenesis. The effect of GnRH administration depends on the size and functional status of the follicles [9]. It induces ovulation of large dominant follicle and luteinization of small follicles [10]. Metabolic hormone such as insulin promotes folliculogenesis as well as steroidogenesis by increasing the concentration of insulin-like growth factor-1 (IGF-1). Administration of insulin enhances the follicular growth which is a prerequisite to GnRH treatment in anoestrus buffaloes [9]. Insulin has also been used to restore the cyclicity in farm animals with different success rate [11,12].

Hence, the present study was undertaken to access the efficacy of GnRH and Insulin alone and in combination in dairy buffaloes under field conditions.

## Materials and Methods

### Ethical approval

This study was carried out after approval by the research committee and Institutional Animal Ethics Committee.

### Selection of experimental animals

A total of hundred true anoestrus non-suckled Murrah and upgraded Murrah buffaloes between first to fifth parity, aged 5 to 10 years and not exhibited any signs of oestrus upto 75 days post partum were selected for the study. The true anoestrus was confirmed by smooth ovaries on gyneco-clinical examinations at 10 days interval and serum progesterone concentration less than 1 ng/ml. All the animals were maintained at standard managemental practices during the entire period of study (1-month). Moreover, only those buffaloes were selected who calved normally in previous calving and had no any gynecological affection such as retained fetal membrane, uterine prolapse, metritis, etc. This study was undertaken in the summer season. The average milk production was 8-14 L/animal per day.

### Treatment groups and schedule

Following deworming with fenbendazole (dose @ 7.5 mg/kg body weight, orally), mineral mixture was given @ 50 g/animal per day for 10 days in all the selected buffaloes before the start of treatment. All the selected buffaloes were divided into four groups, each comprising 25 (n=25). In Group I, buffaloes were treated with a single intramuscular injection of Buserelin, a GnRH analogue @ 20 µg (day 0). Buffaloes of Group II were treated with subcutaneous injection of long-acting biphasic insulin @ 0.25 IU/Kg body weight for 5 consecutive days (day 0-4). Buffaloes of Group III were received subcutaneous injection of biphasic insulin (day 0-4), followed by an intramuscular injection of GnRH on day 5 in the above mentioned doses; whereas buffaloes of Group IV were kept as control.

### Blood sampling and serum progesterone estimation

About 5 ml blood samples were collected aseptically from a jugular vein from all the selected buffaloes before the start of treatment. The serum was separated and kept at −20°C until progesterone estimation. The concentration of progesterone was estimated using 96 wells ELISA kits (DSI S.r.l, Italy).

### Monitoring of animals for fertility response

Following treatment, all the animals were observed twice i.e., early morning and late evening for expression of oestrus signs daily for one month. The time taken for onset of oestrus following withdrawal of treatment was also recorded and analyzed. Animals were bred by natural service at oestrus using fertile buffalo bull. Few buffaloes did not breed because of sub-oestrus, which was confirmed by the presence of corpus luteum on either of the ovaries through rectal palpation. Pregnancy was diagnosed per rectally around 60 days post service. Fertility was recorded and analyzed in terms of oestrus induction rate, time taking for induction of oestrus and conception at induced oestrus.

### Statistical analysis

The data obtained for oestrus induction and conception rate among all the treatment groups were analyzed by Chi-square test, whereas, mean time taken (in days) were analyzed by one-way analysis of variance test, using software package SPSS version 16.0, 2010. [13]

## Results and Discussion

### Oestrus induction

The results indicated higher oestrus induction in insulin plus GnRH (64%) followed by both GnRH and insulin (60% each) treated groups. The oestrus induction in treatment groups found to be significantly higher (p<0.05) than control (Table-1). The oestrus induction was 32% more in insulin-treated than the control group (28 vs. 60%) indicating a beneficial effect of insulin. Similar results have been reported by Kumar *et al*. [12] and Gupta *et al*. [14] in buffalo and by Shukla *et al*. [11] in cattle. Insulin is a metabolic hormone that has a significant role in animal reproduction. It stimulates recruitment of follicles [15] as well granulosa cell proliferation and steroidogenesis by increasing serum IGF-I concentration [16].

**Table-1:** Fertility response to treatments in true anoestrus buffaloes.

Groups	Animals treated	Induction of oestrus		Time taken for onset of oestrus (days)		Animals bred	Animals conceived	
				
	N	N	%	Mean	Range	N	N	%
Group I (GnRH)	25	15	60.00	7.60±0.92^a^	416	12	8	66.66
Group II (Insulin)	25	15	60.00	8.80±0.69^a^	315	15	9	60.00
Group III (Insulin+GnRH)	25	16	64.00	14.43±0.83^b^	821	13	9	69.23
Group IV (Control)	25	7	28.00	20.57±1.69^c^	1222	6	4	66.66

Values with different superscripts in column differ significantly (p<0.05).

Similarly, a good response was observed following GnRH treatment as compared to control group (60% vs. 28%). GnRH is secreted from the hypothalamus in discrete pulsatile bursts, one pulse per 2-6 h [17], which act on anterior pituitary and stimulate synthesis and release of gonadotropic hormone viz. LH and FSH. That is why GnRH has been used to treat the true anoestrus cattle and buffaloes however, response to treatment has been variable ranging from 22% to 87% with mean time interval of 4-29 days [14,18-21]. The variable response may be due to the differential action of GnRH on different stages of follicular development.

Synergistic response has been observed in combined treatment of insulin and GnRH (64%) than the other groups. Others have also reported similar findings using combined therapy of insulin and GnRH [14,22]. Insulin increases the follicular diameter, which is a prerequisite for GnRH treatment to induce oestrus and ovulation in anoestrus buffaloes [9].

### Time taken for onset of oestrus

The mean time taken for the onset of oestrus after the end of treatment was found to be minimum (07.66±0.88 days) in group I, which is significantly lower (p<0.05) than the group III and control group (Table-1). The mean time interval for oestrus induction has also been reported 9.5±3.18 days and 10.5±0.63 days following treatment of insulin plus GnRH [14] and GnRH alone [19], respectively. This may be due to the differential action of GnRH on different stages of follicular development.

### Conception rate

The result indicated higher conception rate in combined treatment of insulin and GnRH as compared to alone insulin and GnRH (69.23% vs. 60.00% and 66.66%) treated group, respectively (Table-1) however, did not differ significantly among treated groups. Higher conception rate in Group III may be due to insulin administration as an increase in IGF-1 concentration in response to insulin treatment enhances the function of corpus luteum and subsequent embryo development [23,24]. The ovulation and conception rate has been reported 100% by Gupta *et al*. [14] but these differences might be due to variation in seasons when the study was undertaken. The results showing higher conception in control but less induction of oestrus thus less number of animals bred, which is not sufficient data for drawing any conclusion. Literature is scant regarding such treatments in buffaloes to compare the results of the present study.

## Conclusion

The findings of the present study indicated better fertility response of insulin pre-treatment to GnRH schedule in true anoestrus buffaloes in terms of oestrus induction and conception rate under field condition. However, satisfactory fertility response has obtained using insulin or GnRH alone. These treatment protocols can be used as per the necessity at farmer’s door.

## Authors’ Contributions

RDP has carried out the present work for his MVSc. thesis work. SNS has supervised and helped in research work and manuscript preparation being an advisor and principle investigator of the project. OPS has also supervised all the activities of research work as advisor. PRK has been associated with the author for entire period of study and helped in the work such as experiments in animals, estimation of progesterone, analysis of data etc. All authors participated in drafting and revision of manuscript. All authors read and approved the final manuscript.

## References

[1] Kodagali S.B (1968). A note on reproductive disorders of farm animals in Saurshtra. Indian J. Vet. Sci. Anim. Husb.

[2] Luktuke S.N, Bhattacharyya A.R, Singh S.K, Khan B.V (1973). Studies on aberration in functional activity of the ovaries in buffaloes. Indian Vet. J.

[3] Kumar P.R, Shukla S.N, Shrivastava O.P, Purkayastha R.D (2013). Incidence of postpartum anoestrus among buffaloes in and around Jabalpur. Vet. World.

[4] Thakor D, Patel D (2013). Incidence of infertility problems in cattle and buffaloes. Dairy Cattle.

[5] Kumar P.R, Shukla S.N, Purkayastha R.D (2013). Economical analysis of the estimated cost of management of anoestrus buffaloes under field conditions using different hormonal and non–hormonal strategies. J. Anim. Health Prod.

[6] Ghuman S.P.S, Singh J, Honparkhe M, Dadarwal D, Dhliwal G.S, Singh S.T (2010). Fate of dominant follicle in summer anoestrus buffaloes. Indian J. Anim. Reprod.

[7] Das G.K, Kumawat B.L, Khan F.A (2013). Ovarian follicular dynamics during estrous cycle and its aberrations during certain reproductive disorders in buffalo. Theriogenology.

[8] Peter A.T, Vos P.L.A, Ambrose D.J (2009). Postpartum anoestrus in dairy cattle. Theriogenology.

[9] Ramoun A.A, Serur B.H, Fattouh El.S.M, Darweish S.A, Abou E1.G.H (2012). Enhancing follicular growth as a prerequisite for GnRH treatment of true anoestrum in buffalo. Anim. Reprod. Sci.

[10] Noakes D.E, Parkinson T.J, England, Gary C.W (2009). Veterinary Reproduction and Obstetrics.

[11] Shukla S.N, Agarwal S.K, Shanker U, Varshney V.P, Majumdar A.C (2005). Ovarian function and restoration of fertility using insulin in acyclic dairy cattle. Indian J. Anim. Sci.

[12] Kumar P.R, Shukla S.N, Shrivastava O.P, Mishra A, Purkayastha R.D (2013). Therapeutic management of true anoestrus in dairy buffaloes (*Bubalus bubalis*) using PMSG with metabolic hormone. Vet. Pract.

[13] SPSS (2010). Statistical Package for Social Sciences.

[14] Gupta V, Thakur M.S, Agrawal R.G, Quadri M.A, Shukla S.N (2010). Effect of pretreatment with Insulin on ovarian and fertility response in true anoestrus buffaloes to Gonadotrophin-Releasing hormone. Buffalo Bull.

[15] Kezele P.R, Nilson E.E, Skinner M.K (2002). Insulin but not insulin like growth factors-1 promotes the primordial to primary follicle transition. Mol. Cell Endocrinol.

[16] Silva J.R.V, Figueiredo J.R, Van den Hurk R (2009). Involvement of growth hormone and insulin-like growth factor system in ovarian folliculogenesis. Theriogenology.

[17] Adsule S.M, Baig M.S, Gade P.R, Khandelwal P.N (2006). GnRH analogues and their role in gynaecology. Antiseptic.

[18] Sirmour S, Nema S.P, Singh B.K, Shukla S.P (2006). Induction of oestrus in delayed pubertal crossbred heifer. Indian J. Anim. Reprod.

[19] Puranik P, Sadasiva Rao K, Solmon Raju K.G (2010). Effect of GnRH, PMSG and Placentrax on reproductive performance of post partum true anoestrus in Murrah buffaloes. Indian J. Anim. Res.

[20] Kumar P.R, Singh S.K, Kharche S.D, Govindaraju C.S, Behera B.K, Shukla S.N, Kumar H, Agarwal S.K (2014). Anoestrus in cattle and Buffalo:Indian perspective. Adv. Anim. Vet. Sci.

[21] McDougall S (2010). Effects of treatment of anestrous dairy cows with gonadotropin-releasing hormone, prostaglandin, and progesterone. J. Dairy Sci.

[22] Singh S.K, Ram H, Naresh R (2007). Induction of oestrus by insulin treatment in anoestrus cattle. XXIII Annual Convention of ISSAR and National Symposium.

[23] Palma G.A, Muller M, Brem G (1997). Effect of insulin-like growth factor I (IGF-I) at high concentrations on blastocyst development of bovine embryos produced *in vitro*. J. Reprod. Fertil.

[24] Baithalu R.K, Singh S.K, Gupta C, Raja A.K, Saxena A, Agarwal S.K (2013). Insulin stimulates progesterone secretion to a greater extent than LH in early pregnant buffalo luteal cells cultured *in vitro*. Anim. Reprod. Sci.

